# Case Report: Identification of a novel hemizygous missense *RPL10* gene variant in two unrelated patients

**DOI:** 10.3389/fped.2025.1570911

**Published:** 2025-08-08

**Authors:** Lixia Xu, Ke Wu, Yan Cong

**Affiliations:** ^1^ Obstetrics Department, Quzhou Maternal and Child Health Care Hospital, Quzhou, Zhejiang, China; ^2^ Laboratory of Prenatal Diagnosis, Quzhou Maternal and Child Health Care Hospital, Quzhou, Zhejiang, China; ^3^ Rehabilitation Department, Yiwu Maternity and Children Hospital (Yiwu Hospital of Children's Hospital Zhejiang University School of Medicine), Yiwu, Zhejiang, China

**Keywords:** intellectual disability, *RPL10* gene, psychomotor development delay, functional analysis, loss-of-function

## Abstract

The X-linked syndromic intellectual developmental disorder-35 (MRXS35; OMIM#300998) is caused by variants in the *RPL10* gene (OMIM*312173) on chromosome Xq28. Patients with MRXS35 mainly present with intellectual disability (ID), psychomotor development delay, speech delay, short stature, craniofacial anomalies, hypotonia, seizures, gastrointestinal problems, genitourinary anomalies, cardiac anomalies, eye defects, and hearing loss. Herein, we are the first to report two unrelated Chinese patients with the same novel hemizygous missense *RPL10* gene variant. Two male patients from two different families were admitted to the hospital for genetic counseling. In the first months of life, both newborns presented with congenital laryngeal stridor, feeding difficulties, neonatal pneumonia, neonatal hypoglycemia, dysmorphic features, and bilateral cryptorchidism. At his last clinical evaluation at 9 years of age, case II presented with ID, speech delay, short stature, and craniofacial anomalies. Whole-exome sequencing identified the same hemizygous missense *RPL10* gene variant (NM_006013.5:c.347G>A, p.Arg116Gln) in each patient, inherited from their respective mothers. The functional analysis of this variant *in vitro* demonstrated that this missense *RPL10* gene variant (c.347G>A) reduced the mRNA expression of the *RPL10* gene, thereby decreasing synthesis of the RPL10 protein. Our *in vitro* functional analysis indicated a loss-of-function effect of *RPL10* gene variants.

## Introduction

The *ribosomal protein L10* (*RPL10*) gene (OMIM*312173) is located on chromosome Xq28 and encodes a highly conserved component of the large ribosome subunit (60S) that plays a crucial role in protein synthesis (1). A high expression of RPL10 protein was observed in the hippocampus of mice (2) and vertebrate epiphyseal cartilage (3). Disease-causing *RPL10* variants have been found to be responsible for X-linked recessive syndromic intellectual developmental disorder-35 (MRXS35; OMIM#300998). Susceptibility to X-linked autism-5 (AUTSX5; OMIM#300847) is also associated with variants in the *RPL10* gene (4, 5). In 2014, Brooks et al. first reported that the transmission pattern of MRXS35 in a family was consistent with X-linked recessive inheritance (6). Patients with MRXS35 mainly present with intellectual disability (ID), psychomotor development delay, speech delay, short stature, craniofacial anomalies, hypotonia, seizures, gastrointestinal problems, genitourinary anomalies, cardiac anomalies, eye defects, and hearing loss. In the past decade (2014∼2024), only seven missense *RPL10* gene variants have been identified in the 19 previously reported male patients. Herein, we describe two unrelated Chinese children with the same novel hemizygous missense *RPL10* gene variant, which expands the mutational spectrum of the *RPL10* gene and the clinical spectrum of MRXS35.

## Materials and methods

### Clinical features

#### Case I

Patient Ⅰ is the only child of healthy, non-consanguineous parents. During the antenatal period, fetal growth delay was reported. His mother had a history of gestational diabetes. He was born at term with a weight of 2,650 g (*Z*-score: −1.53), a length of 47.2 cm (*Z*-score: −1.42), and a head circumference of 28.3 cm (*Z*-score: −4.85). He was noted to have dysmorphic features, including microcephaly, microretrognathia, protruding ears, nasal bridge collapse, and a high-arched palate. Furthermore, adducted thumbs, a single transverse palmar crease, and bilateral cryptorchidism were found. At his last clinical evaluation at 2 months, the newborn had a weight of 2,650 g (−1 SD), a length of 47.2 cm (−1 SD), and a head circumference of 26.3 cm (−3 SD). He also presented with feeding difficulties, congenital laryngeal stridor, neonatal pneumonia, and neonatal hypoglycemia.

#### Case II

Patient II was the only child of healthy, non-consanguineous parents. The antenatal period was normal. He was born after 39 weeks of uncomplicated gestation with a weight of 2,540 g (*Z*-score: −1.80), a length of 47.9 cm (*Z*-score: −1.05), and a head circumference of 29.8 cm (*Z*-score: −3.67). In the first months of life, the newborn presented with bilateral cryptorchidism, congenital laryngeal stridor, feeding difficulties, neonatal pneumonia, and neonatal hypoglycemia. However, adducted thumbs and a single transverse palmar crease were not found in this case. He started walking independently at the age of 22 months. At the age of 6 years, he had a general quotient (GQ) of 17.5 (the patient’s raw scores for the five subscales were lower than the first percentile, and the language score was not completed as the child could not cooperate). At the last clinical evaluation at the age of 9 years, he was still non-verbal. His weight was 20.0 kg (*Z*-score: −2.75), and his length was 120.5 cm (*Z*-score: −2.17). He had dysmorphic features, including a small forehead, microretrognathia, protruding ears, and a high-arched palate. He was diagnosed with growth retardation and intellectual disability (Table 1).

**Table 1:** Summary of the clinical phenotypes of cases I and II.

Case	Age at the last clinical evaluation	Prenatal phenotypes	Postnatal phenotypes
Case I	2 months	Fetal growth delay	Development delay, feeding difficulties, congenital laryngeal stridor, neonatal pneumonia, neonatal hypoglycemia, craniofacial anomalies, and cryptorchidism
Case II	9 years	Unremarkable	Feeding difficulties, congenital laryngeal stridor, neonatal pneumonia, neonatal hypoglycemia, intellectual disability, psychomotor development delay, speech delay, short stature, craniofacial anomalies, and cryptorchidism

Between December 2020 and December 2024, these two male patients were referred to the Quzhou Maternal and Child Health Care Hospital and Yiwu Maternity and Child Health Care Hospital, respectively, for genetic counseling. Due to their multiple congenital anomalies, whole-exome sequencing (WES) was recommended and conducted. The study was approved by the Medical Ethics Committee of Quzhou Maternal and Child Health Care Hospital, and written informed consent was obtained from the children’s parents.

### Whole-exome sequencing

Trio-based whole-exome sequencing (trio-WES) was performed on genomic DNA (gDNA) from peripheral blood samples of the patients and their parents. The xGen™ Exome Research Panel v2 (designed by Integrated DNA Technologies, Coralville, IA, USA) was used for WES. Quality control (QC) of the DNA library was performed using an Agilent 2100 Bioanalyzer System (Agilent, Santa Clara, CA, USA). DNA nanoball (DNB) preparations of clinical samples were sequenced using the ultra-high-throughput DNBSEQ-T7 platform (MGI, Shenzhen, China) with a paired-end 150 nt strategy, following the manufacturer's protocol.

### Bioinformatic analysis

The sequencing data were analyzed according to our in-house (Chigene Translational Medicine Research Center) procedures. Adapters and low-quality reads were removed, and then data quantity and quality were analyzed. The trimmed reads were then mapped to the University of California at Santa Cruz (UCSC) GRCh37/hg19 reference genome using Burrows–Wheeler Aligner (BWA) software. The Genome Analysis ToolKit (GATK) software was used for single-nucleotide polymorphisms (SNPs) and short (<50 bp) insertion/deletion (indel) calling. The SAMtools and Picard software packages were used to generate clean binary alignment map (BAM) data by removing duplicate data. Variants were annotated for analysis using the single-nucleotide polymorphism database (dbSNP), the gnomAD exomes database, and the Chigene Translational Medicine Research Center’s in-house minor allele frequency (MAF) database. Pathogenicity prediction tools, including the Rare *Exome* Variant Ensemble Learner (REVEL) and AlphaMissense, were used to predict the possible effect of the variants.

### Functional analysis of the *RPL10* variant *in vitro*

Mutant *RPL10* gene pcDNA3.1(+)-MUT-3xFlag and wild-type pcDNA3.1(+)-WT-3xFlag mammalian expression vectors were constructed. Human embryonic kidney (HEK)-293 T cells were transfected with expression vectors in two tubes (one for the mutant expression vectors and one for the wild-type expression vectors). The constructed expression vectors were verified using Sanger sequencing. Then, 6 h after the transfection, we gently removed the lipofectamine-containing medium, replaced the medium with Dulbecco's modified Eagle medium (DMEM) containing 5% fetal bovine serum (FBS), and incubated the slide at 37°C in a 5% CO_2_ incubator until day 2. On day 3, the samples were collected and used in a reverse transcription-polymerase chain reaction (RT-PCR) to quantify the *RPL10* mRNA expression and a Western blot (WB) assay to quantify the protein expression. Each cell line underwent three independent replicates of the RT-PCR assay. The RT-PCR and Western blotting experiments were conducted in accordance with the manufacturer's instructions.

## Results

### Genetic analysis

The WES identified the same hemizygous missense *RPL10* gene variant [NM_006013.5:c.347G>A, p.Arg116Gln; chrX:g.154400481G>A (hg38)] in patients I and II. The Sanger sequencing also confirmed that the same hemizygous variant was derived from their respective heterozygous carrier mothers, and their respective fathers were normal. The allele frequency of c.347G>A has not been registered in population databases (1000 Genomes Project, gnomAD, and GenomeAsia) (PM2_Supporting). It has been reported in ClinVar (RCV001333474, RCV004789530) as a variant of uncertain significance (VUS). The PhastCons100way conservation score was 1.000 (range of 0–1; the greater the score, the more conserved the site). This variant causes a missense change involving the alteration of a conserved nucleotide. The *in silico* predictive algorithm result for this variant showed that the missense variant was moderately deleterious (REVEL score of 0.75 > 0.64; AlphaMissense score of 0.998 > 0.869) (PP3_Moderate). As per the interpretation guidelines by the American College of Medical Genetics and Genomics (7), this novel variant was classified as VUS (PP4; PM2; PP3). We have submitted this variant to a public database [Leiden Open Variation Database (LOVD)], and the accession number(s) can be found at the following URL: https://databases.lovd.nl/shared/individuals/00464560.

### Functional analysis of the VUS allele

**Figure 1:**
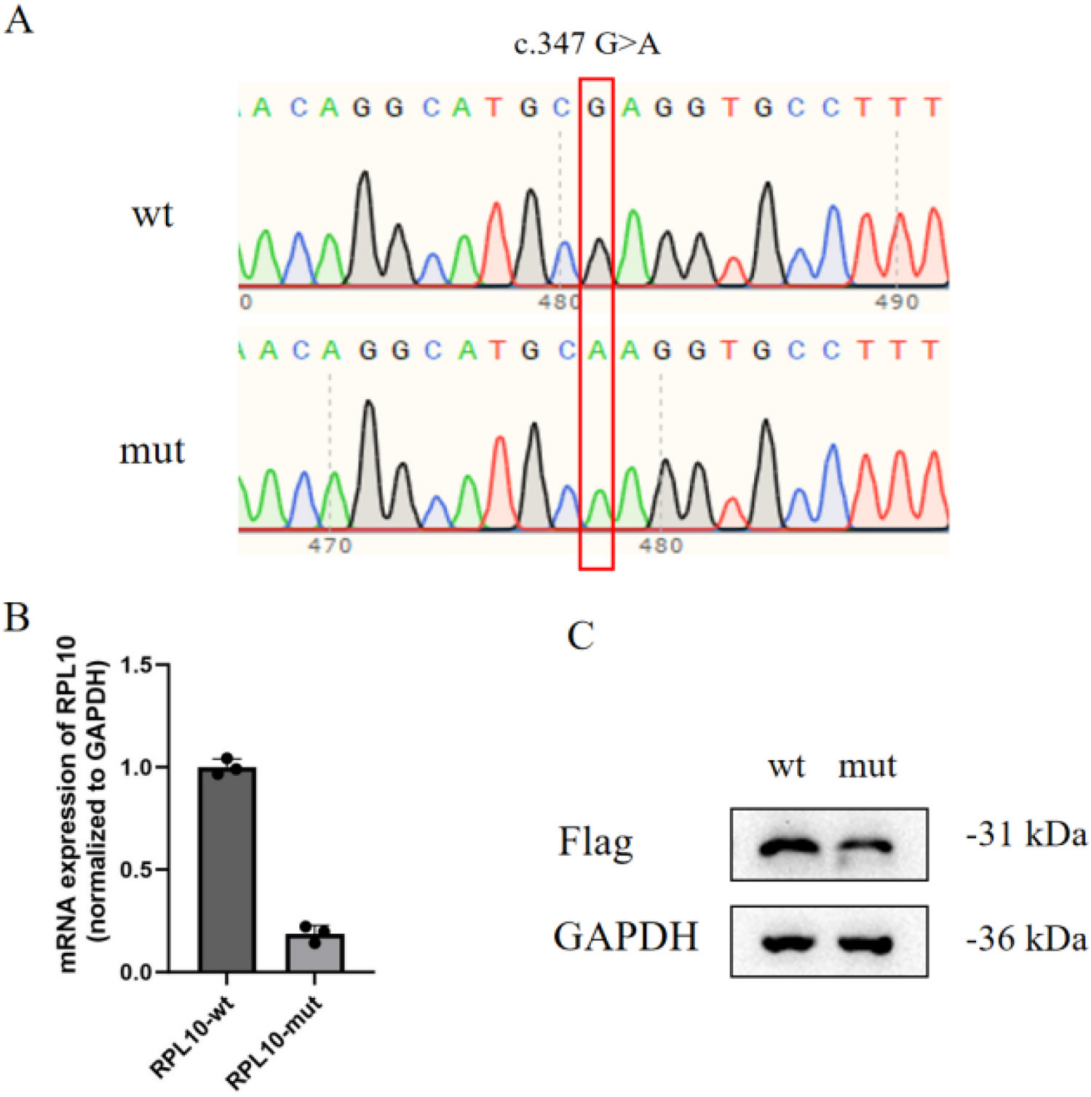
Functional analysis of the VUS allele *in vitro*. (**A**) Constructed mutant and wild-type mammalian expression vectors (the variant is marked by the red box). (**B**) The relative mRNA expression of the *RPL10* gene (normalized to the control gene, *GAPDH*) indicates that the level of relative mRNA expression in the *RPL10*-mut cell line is significantly lower than in the *RPL10*-wt cell line. Each cell line underwent three independent replicates of the RT-PCR assay (*p* < 0.01). Statistical analysis data are provided in Supplementary Data 1. (**C**) The Western blot analysis shows reduced formation of RPL10 protein in the *RPL10*-mut cell line (the RPL10 protein gray-scale value in *RPL10*-wt was 157,808; the RPL10 protein gray-scale value in *RPL10*-mut was 108,767). One technical replicate was performed. The WB band gray-scale values (each wt/mut lane) are provided in Supplementary Data 2.

A functional analysis of the *RPL10* gene variant (NM_006013.5) c.347G > A was performed *in vitro*. Mutant *RPL10* gene pcDNA3.1(+)-MUT-3xFlag and wild-type pcDNA3.1(+)-WT-3xFlag mammalian expression vectors were constructed. The results of the Sanger sequencing of the mutant and wild-type expression vectors confirmed that the construction of the vectors was successful (Figure 1A). Then, HEK-293 T cells were transfected with the expression vectors. The RT-PCR assay revealed that the mRNA expression of the *RPL10* gene variant (c.347G>A) was significantly lower than that of the wild-type mRNA (Figure 1B). Each cell line underwent three independent replicates of the RT-PCR assay. The Western blot analysis showed reduced formation of the RPL10 protein in the *RPL10*-mut cell line (Figure 1C). We speculate that the reduction in RPL10 protein may have been a result of the variant-induced reduction in mRNA expression. Due to the functional experimental data, this variant was reclassified as likely pathogenic.

## Discussion

The *RPL10* gene (OMIM*312173) is located on chromosome Xq28. The transcript of *RPL10* (NM_006013.5) has seven exons with a transcript length of 2,164 base pairs. The RPL10 protein is a 214-amino-acid ribosomal protein that is a critical component of the 60S ribosome subunit. As part of the ribosome, RPL10 plays structural and functional roles in the formation of actively translating ribosomes. Variants or dysregulation of RPL10 have been linked to neurodevelopmental disorders, such as MRXS35 (OMIM#300998) and AUTSX5 (OMIM#300847), as well as certain cancers, including T-cell acute lymphoblastic leukemia (T-ALL) (8) and multiple myeloma (9).

These two patients are the first reported cases of MRXS35 in China. The WES identified the same hemizygous missense *RPL10* gene variant (NM_006013.5:c.347G>A, p.Arg116Gln) in these two unrelated patients. This variant has not been previously reported in the literature. These two patients had similar clinical presentations, overlapping with some clinical phenotypes of MRXS35 such as ID, psychomotor development delay, speech delay, short stature, craniofacial anomalies, and cryptorchidism. Some of the patients’ phenotypes have not been reported, such as congenital laryngeal stridor, feeding difficulties, neonatal pneumonia, and neonatal hypoglycemia. The symptoms of neonatal hypoglycemia and hypoglycemia (caused by bacteria) were transient, and both cases showed significant improvement with appropriate interventions. Some MRXS35 phenotypes, such as hypotonia, seizures, gastrointestinal problems, cardiac anomalies, eye defects, and hearing loss, were not observed in these two unrelated patients.

Currently, there are only 14 reported patients with pathogenic *RPL10* gene variants associated with MRXS35 (Supplementary Table S1). They have mainly presented with ID, psychomotor development delay, short stature, craniofacial anomalies, hypotonia, seizures, gastrointestinal problems, genitourinary anomalies, cardiac anomalies, eye defects, and hearing loss. The functional analyses of two missense *RPL10* gene variants (p.L206M and p.H213Q) identified in four patients with autism suggested that these variants decreased the translational capacity of ribosomes, ultimately impacting synaptic plasticity and neurodevelopment (4). Another functional study showed that knockdown of the *rpl10* gene in zebrafish decreased head size in developing morphant embryos and increased apoptosis in the brain. Coinjection of *rpl10* translation blocking-morpholinos with mRNA harboring p.K78E failed to rescue the morphant phenotype. These data demonstrated that c.232A > G (p.K78E) is a loss-of-function variant (6). Similarly, our *in vitro* functional analysis indicated that the pathogenic mechanisms of *RPL10* gene variants may be a loss-of-function effect.

We used a splicing prediction tool (SpliceAI) (10) to evaluate whether the variant in this study was involved in splicing events. The SpliceAI score of the variant in this study was 0.010 (SpliceAI score ≤ 0.1), which indicated a moderate level of evidence for non-spliceogenicity (BP4) (11). Several *in silico* tools (AlphaMissense, PrimateAI, REVEL, and MetaRNN) predicted a pathogenic outcome for the variant in this study. When compared to c.232A>G (p.K78E) reported by Brooks et al (6), both variants are missense, and the *in silico* tools (AlphaMissense, PrimateAI, REVEL, and MetaRNN) also predicted a pathogenic outcome for c.232A>G (p.K78E). The SpliceAI score of c.232A>G (p.K78E) was 0.

The RPL10 protein is a critical component of the 60S ribosome subunit, which includes approximately 49 different proteins and 3 RNA molecules (28S, 5.8S, and 5S) (12). According to the results of the functional studies performed by Klauck et al. (4) and Brooks et al. (6), we speculate that the Arg116Gln substitution may disrupt the interaction between the RPL10 protein and RNA, shortening the translation rate and consequently significantly reducing RPL10 protein expression.

In conclusion, the hallmark phenotypes of MRXS35 are intellectual disability, growth retardation, speech delay, microcephaly, craniofacial anomalies, seizures, hypotonia, and cryptorchidism. These two patients broaden the clinical phenotypes of MRXS35. Although our *in vitro* functional analysis indicated that *RPL10* gene variants have a loss-of-function effect, further in-depth studies on the pathogenic mechanisms of *RPL10* gene variants are still needed to provide a more comprehensive understanding of the diagnosis and treatment of MRXS35.

## Data Availability

The original contributions presented in the study are included in the article/Supplementary Material, further inquiries can be directed to the corresponding author.
