# Supplementary material for: Case Report: Identification of a novel hemizygous missense RPL10 gene variant in two unrelated patients
Source: Front Pediatr. 2025 Aug 8;13:1570911. doi: 10.3389/fped.2025.1570911 (PMC12371927; doi:10.3389/fped.2025.1570911)
Supplement: Supplementary file 1 [file Table1.docx]

| Supplementary table 1: male patients with *RPL10* gene variants | | | | |
| --- | --- | --- | --- | --- |
| Cases | *RPL10* variants  (NM 006013.3) | Prenatal presentations | Postnatal presentations | Brain MRI |
| Zanni et al(2015)[^[[1]](#endnote-0)^] | c.191C>T (p.A64V) associated with MRXS35 | Placental insufficiency and poor fetal growth. | Neonatal hypotonia, moderate delay in psychomotor development, and growth retardation, walked at 4 years of age with an ataxic gait, retinitis pigmentosa. At the age of 5 years, he was surgically operated for cryptorchidism and inguinal hernia. Body X-ray performed at the age of 14 years showed spondylo-epiphyseal dysplasia (SED), scoliosis, and osteoporosis. Hypothyroidism, gowth retardation and minor cranio-facial anomalies (dolicocephaly, long and flat philtrum, microretrognathia, protruding ears with absent anthelix). | Cerebellar hypoplasia, predominantly in the vermis |
|  |  | regular pregnancy | sat at 14-15 months, started to walk at 4 years of age with an ataxic gait, speech delay, cryptogenic focal epilepsy SED, dorso-lumbar scoliosis, similar cranio-facial features | cerebellar hypoplasia |
| Brooks et al(2014)[^[[2]](#endnote-1)^] | c.232A>G (p.K78E) associated with MRXS35 | polyhydramnios at 27 weeks gestation | born at 35 weeks, microcephaly, Seizures, Severe growth retardation, hypotonia, craniofacial defects (prognathic, thin upper lip, asymmetric dilation of the right temporal horn), right cryptorchidism, mild camptodactyly; proximal partial syndactyly; gastroesophageal reflux disease, recurrent fever/pneumonia, negative immune work-up, sacral lipoma | NA |
|  | c.232A>G (p.K78E) associated with MRXS35 | NA | born at 38 weeks. microcephaly, seizures, severe growth retardation, hypotonia, prognathic, dental crowding, hypospadias, right cryptorchidism,tapered fingers, ASD, pulmonary artery stenosis, laryngomalacia, self-abusive behaviors, recurrent fevers/infection in childhood, chronic GERD, bilateral hearing loss, contractures of knees and ankles | NA |
|  | c.232A>G (p.K78E) associated with MRXS35 | NA | born at 37 weeks. microcephaly, seizures, severe growth retardation, hypotonia. prognathic, protuberant ears, branchial cleft cyst; hypospadias, cryptorchidism; unilateral simian crease, VSD, recurrent fevers/infection in childhood, GERD, mild sensorineural hearing loss, movement disorder, nonverbal | NA |
| Cappuccio et al (2022)[^[[3]](#endnote-2)^] | c.95G>T (p.Arg32Leu) associated with MRXS35 | unremarkable | cesarean section at 37 weeks of gestation; birth weight was 2900 g (7th centile), growth retardation, delayed language skill, microcephaly, cryptorchidism brachycephaly, bilateral palpebral ptosis, epicanthus, bilateral crumpled helix, depressed nasal root, long and flat philtrum, prognathism, bifid uvula, small and spaced teeth, brachydactyly and bilateral clinodactyly of the fifth toe, hypoplasia of the middle phalanx of the fifth finger retinal anomalies | mild hypoplasia of the lower portion of the cerebellar vermis |
|  | c.95G>T (p.Arg32Leu) associated with MRXS35 | NA | cesarean section after 37 weeks of gestation; microcephaly, growth retardation, delayed language skill, hyperactivity and self-injurious behaviors, bilateral cryptorchidism, long face with narrow and down-slanting palpebral fissures, epicanthus, and esotropia of the left eye, long and flat philtrum, thin upper lip, enamel hypoplasia. shawl scrotum and bilateral syndactyly of second and third toe mild. mitral prolapse and tricuspid prolapse with regurgitation without aortic root dilatation retinal anomalies | normal |
|  | c.95G>T (p.Arg32Leu) associated with MRXS35 | NA | born after 37 weeks of uncomplicated gestation; microcephaly, growth retardation, delayed language skill, long face with narrow and down-slanting palpebral fissures, long and flat philtrum, and thin upper lip; bilateral syndactyly of second and third toe; mild mitral prolapse and tricuspid regurgitation retinal anomalies | NA |
|  | c.95G>T (p.Arg32Leu) associated with MRXS35 | unremarkable | emergency cesarean section because of abnormal cardiotocography after 39 weeks of gestation; microcephaly, seizures, growth retardation, hypotonia, delayed language skill , recurrent ear infections and obstructive sleep disorder. dysmorphic features including long face with flat profile, sparse eyebrows, internal epicantal folds, full eyelids, narrows palpebral fissures, Cupid's bow upper lip, brachydactyly, and bilateral clinodactyly of the fifth fingers of hands and toes, retinal anomalies | small corpus callosum |
| Thevenon et al (2015)[^[[4]](#endnote-3)^] | c.481G > A(p.Gly161Ser) associated with MRXS35 | hydramnios | born at 35 weeks of gestation. language skills were delayed, bacterial meningitis and a single febrile seizure nonspecific white matter anomalies. Bilateral cryptorchidism and dorsal hirsutism a stocky build, short fingers, bilateral clinodactyly of the fifth fingers, single palmar creases, and 2-3 partial cutaneous syndactyly of the toes. Facial features included prominent ears, a broad nasal ridge, bilateral epicanthus, and a small mouth, cryptorchidism, mild ataxia | mild posterior periventricular white matter hypersignals and significant dilation of the Virchow-Robin spaces |
|  | c.481G >A (p.Gly161Ser) associated with MRXS35 | hydramnios | hypotonia and feeding difficulties, possibly secondary to gastroesophageal reflux, language skills were delayed. growth retardation tremor and ataxia a single febrile seizure, myopia. Microcephaly, large prominent ears, wide hands with bilateral single palmar creases, and wide feet. Cerebellar syndrome | NA |
|  | c.481G >A (p.Gly161Ser) associated with MRXS35 | NA | No microcephaly(22 year), growth retardation, delayed language skill, ID, cryptorchidism, mild anal anteposition, delayed puberty, severe myopia | NA |
|  | c.481G>A(p.Gly161Ser) associated with MRXS35 | Mild hydramnios | No microcephaly (16 year), growth retardation, delayed language skill, ID, small testes, severe myopia | NA |
| Bourque et al(2018) [^[[5]](#endnote-4)^] | c.232A>G (p.K78E) associated with MRXS35 | unremarkable | spontaneous vaginal delivery at 36 weeks’ gestation; lethargy and poor feeding as a neonate; microcephaly, delayed language skill, growth retardation, long face with broad forehead; palpebral fissures; high arched, widely spaced teeth, prominent jaw; aggressive and self-injurious behaviour (head banging, hand biting, chewing, spitting); ataxic gaits, epilepsy at the age of 6 years | NA |
| Klauck et al (2006)[^[[6]](#endnote-5)^] | c.616C > A (p.L206M) associated with ASD | intermittent bleeding  between 14th and 20th week | Birth followed at 31st week gestation through cesarean section, left-side hemiplegia, epilepsy at the age of 1 years. **ASD**, delayed language skill, growth retardation | NA |
|  | c.616C > A (p.L206M) associated with ASD | intermittent bleeding  between 14th and 20th week | Birth followed at 31st week gestation through cesarean section, language skills were delayed, growth retardation, severe hyperactivity, ASD | NA |
|  | c.639C > G (p.H213Q) associated with ASD | NA | normal early childhood development, language delay, ASD, ID | NA |
|  | c.639C > G (p.H213Q) associated with ASD | NA | language delay, ASD, ID | NA |
| Chiocchetti et al (2011)[^[[7]](#endnote-6)^] | c.639C > G (p.H213Q) associated with ASD | NA | language delay, ASD, ID | NA |
| patient Ⅰ | c.347G>A (p.Arg116Gln) associated with MRXS35 | fetal growth delay | At the age of two months, the newborn presented with feeding difficulties, congenital laryngeal stridor, neonatal pneumonia and neonatal hypoglycemia, dysmorphic features including microcephaly, microretrognathia, protruding ears, nasal bridge collapse and high-arched palate, adducted thumbs, the single transverse palmar crease, cryptorchidism | Normal |
| patient Ⅱ | c.347G>A (p.Arg116Gln) associated with MRXS35 | unremarkable | congenital laryngeal stridor, feeding difficulties, neonatal pneumonia and neonatal hypoglycemia; growth retardation, language skills were delayed, ID. | Normal |
| Abbreviations: ASD, atrial septal defect; GERD, gastroesophageal reflux disease; SD, standard deviations; VSD, ventricular septal defect, NA, not available | | | | |

**References**

1. [] Zanni G, Kalscheuer VM, Friedrich A, et al. A Novel Mutation in RPL10 (Ribosomal Protein L10) Causes X-Linked Intellectual Disability, Cerebellar Hypoplasia, and Spondylo-Epiphyseal Dysplasia. Hum Mutat. 2015;36(12):1155-1158. doi:10.1002/humu.22860 [↑](#endnote-ref-0)
2. [] Brooks SS, Wall AL, Golzio C, et al. A novel ribosomopathy caused by dysfunction of RPL10 disrupts neurodevelopment and causes X-linked microcephaly in humans. Genetics. 2014;198(2):723-733. doi:10.1534/genetics.114.168211 [↑](#endnote-ref-1)
3. [] Cappuccio G, De Bernardi ML, Morlando A, et al. Postnatal microcephaly and retinal involvement expand the phenotype of RPL10-related disorder. Am J Med Genet A. 2022;188(10):3032-3040. doi:10.1002/ajmg.a.62911 [↑](#endnote-ref-2)
4. [] Thevenon J, Michot C, Bole C, et al. RPL10 mutation segregating in a family with X-linked syndromic Intellectual Disability. Am J Med Genet A. 2015;167A(8):1908-1912. doi:10.1002/ajmg.a.37094 [↑](#endnote-ref-3)
5. [] Bourque DK, Hartley T, Nikkel SM, et al. A de novo mutation in RPL10 causes a rare X-linked ribosomopathy characterized by syndromic intellectual disability and epilepsy: A new case and review of the literature. Eur J Med Genet. 2018;61(2):89-93. doi:10.1016/j.ejmg.2017.10.011 [↑](#endnote-ref-4)
6. [] Klauck SM, Felder B, Kolb-Kokocinski A, et al. Mutations in the ribosomal protein gene RPL10 suggest a novel modulating disease mechanism for autism. Mol Psychiatry. 2006;11(12):1073-1084. doi:10.1038/sj.mp.4001883 [↑](#endnote-ref-5)
7. [] Chiocchetti A, Pakalapati G, Duketis E, et al. Mutation and expression analyses of the ribosomal protein gene RPL10 in an extended German sample of patients with autism spectrum disorder. Am J Med Genet A. 2011;155A(6):1472-1475. doi:10.1002/ajmg.a.33977 [↑](#endnote-ref-6)
